# Editorial: An Update on Brassinosteroids: Homeostasis, Crosstalk, and Adaptation to Environmental Stress

**DOI:** 10.3389/fpls.2021.673587

**Published:** 2021-04-23

**Authors:** Damian Gruszka, Andrzej Bajguz, Qian-Feng Li, Shamsul Hayat, Mats Hansson, Xuelu Wang, Jianming Li

**Affiliations:** ^1^Faculty of Natural Sciences, Institute of Biology, Biotechnology and Environmental Protection, University of Silesia, Katowice, Poland; ^2^Faculty of Biology, University of Bialystok, Bialystok, Poland; ^3^Jiangsu Key Laboratory of Crop Genomics and Molecular Breeding, College of Agriculture, Yangzhou University, Yangzhou, China; ^4^Department of Botany, Faculty of Life Sciences, Aligarh Muslim University, Aligarh, India; ^5^Department of Biology, Lund University, Lund, Sweden; ^6^Center of Integrative Biology, College of Life Science and Technology, Huazhong Agricultural University, Wuhan, China; ^7^Department of Molecular, Cellular and Developmental Biology, University of Michigan, Ann Arbor, MI, United States

**Keywords:** phytohormones, biosynthesis, crosstalk, homeostasis, signaling, environmental stress, brassinosteroids

Over the last three decades, there have been significant advances in the understanding of brassinosteroid (BR) biosynthesis and signaling, particularly in the model plant species *Arabidopsis thaliana*. BRs regulate a variety of morphogenetic and physiological processes throughout plant life. Notably, BR biosynthesis and signaling are interconnected with the signaling pathways of other phytohormones and environmental stresses. Gathering knowledge about these aspects in monocot and dicot crops is of particular importance as it may allow modulation of these processes and enable the development cultivars better adapted to ongoing climate change. This Research Topic, providing *An Update on Brassinosteroids: Homeostasis, Crosstalk, and Adaptation to Environmental Stress* is aimed at introducing the latest findings in the regulation of BR metabolism, the interconnection of the BR signalosome with phytohormonal and stress signaling pathways, and the BR-mediated adaptation of plants to environmental conditions. The Research Topic includes five reviews and one original research article.

As far as the BR biosynthesis pathway is concerned, Bajguz et al. provided a comprehensive review of the complicated process. The authors presented a description of the BR biosynthesis and their sterol precursors. BR biosynthesis leads to the production of the C_27_-, C_28_-, or C_29_-type BRs. Thus, the authors described the early steps of the process that are common for each type and later presented the steps which differentiate the BR biosynthesis pathways. The interconnections between the distinct BR biosynthesis pathways were also described. An important part of this review is focused on the BR biosynthesis inhibitors because they are useful tools for investigating the biosynthetic pathways and for manipulating the BR content in crops.

Another review article by Wei and Li presented the recent advance in elucidating mechanisms that control the BR homeostasis in reaction to both internal and external cues. Understanding this process is of particular importance, as BRs control plant growth and development in a dose-dependent manner, and a fine-tuning of BR homeostasis is critical for optimal BR functions. This article converges with the above-mentioned review and places aspects of BR biosynthesis in a broader, regulatory context. The review presented the regulation of BR biosynthesis and catabolism by hormonal and environmental cues in Arabidopsis but also in rice and other crop species. It is of significant importance, as elucidating the mechanisms regulating the BR homeostasis may allow the development of new, high yield crops *via* manipulating BR contents.

A series of articles in this Research Topic described the role of BRs in the regulation of plant reaction to environmental stresses. The first article of this Research Topic by Ramirez and Poppenberger discusses the role of BR in the regulation of cold stress tolerance. Interestingly, in comparison with gibberellins, BRs improve cold stress tolerance with fewer trade-offs in terms of growth and yield. The authors indicated that in addition to improving basal tolerance, BRs contribute to acquired freezing tolerance in Arabidopsis. It involves the molecular and biochemical changes induced by low, but non-freezing temperatures. This adaptation may result from the fact that BRs are involved in cell wall remodeling mechanisms. It was also reported that BRs can increase lignin accumulation. The article comprehensively reviewed biochemical and cellular adaptations during plant reaction to the cold stress, with an emphasis on the BR influence on the cold stress perception and signaling.

Another review article by Kour et al. described the role of BRs in the regulation of plant reaction to heavy metal stress. BRs reduce the uptake of heavy metals by altering cell membrane permeability. They also induce antioxidant enzymes which scavenge the reactive oxygen species that accumulate within the cell in reaction to the stress. BRs alleviate heavy metal toxicity by increasing the concentration of potassium and sodium ions, proline, antioxidants, and osmolytes. The article includes a review of various experiments in which crop plants were exposed to heavy metal stress and were simultaneously treated with exogenous BRs. The article also presented an emerging view of interhormonal crosstalk (mostly involving BR, auxin, and cytokinin) and the interplay of BR-polyamines, which regulate reaction to stress.

The molecular mechanisms underlying the regulatory role of BRs in the modulation of plant growth and stress responses are described in a review article by Kono and Yin. The article described the functions and regulation of the BES1/BZR1 transcription factors which are key modulators of the BR-dependent gene expression. Moreover, they constitute hubs interconnecting the BR signaling with the response pathways of other phytohormones and environmental cues. Interestingly, SUMOylation appears to have an opposite effects on the functions of the two (highly similar) transcription factors. It is postulated that the opposite effects result from distinct SUMOylation target sites in each transcription factor. Moreover, the SUMOylation of BZR1 is regulated by salt stress, and the BR and salt-stress signaling pathways interact at multiple targets. The review also described the involvement of the BES1/BZR1 transcription factors in the crosstalk between the BR, jasmonic acid, auxin, and UV-B signaling pathways during plant morphogenesis and reaction to environmental stresses.

The interconnection between BR signaling and salt-stress response is also explored in the original research article by Dong et al. The article describes the development of the mutant alleles of *SERK2* (involved in the BR signaling) by the CRISPR/Cas9 method. The knockout of *SERK2* resulted in a compact plant stature but increased grain size. The *serk2* mutants showed an increased salt sensitivity which seemed to be independent of ABA. On the other hand, overexpression of the *SERK2* gene enhanced salt tolerance without a negative effect on plant architecture. Interestingly, *SERK2* positively regulates salt tolerance, however, it is suppressed by salinity at the transcription level. On the contrary, the SERK2 protein accumulation is greatly induced by stress. Generally, *SERK2* could be a valuable target for engineering plant architecture and salt-stress tolerance.

This Research Topic provides important updates on BR homeostasis and functions. However, several aspects such as the involvement of BRs in the epigenetic and small RNA-mediated regulation of gene expression require further research and elucidation.

## Author Contributions

DG wrote the manuscript. All authors revised the manuscript and gave final approval for publication.

## Conflict of Interest

The authors declare that the research was conducted in the absence of any commercial or financial relationships that could be construed as a potential conflict of interest.

